# Monthly Summary

**Published:** 1899-11

**Authors:** 


### Monthly Summary.

COMBINATION FILLINGS.—About fifteen years ago, I began to mix zinc and amalgam in filling teeth, first, mixing the powder and amalgam with the fluid, afterwards using Dr. Spooner's method, as described in August "Items of Interest." I desire to give the result of my experience for the benefit of the profession and not by way of criticism.

My experience with zinc and amalgam mixed together is that the zinc will wear out and leave the amalgam prominent, thus giving a rough surface to the filling.

One-third amalgam and two-thirds zinc will cause a tooth with deep decay to become much discolored, just what we should avoid in the bicuspids or any of the anterior teeth.

It is a good filling to support frail walls, and will wear much better than zinc. If Dr. Spooner will use the zinc and amalgam, and in finishing cover it with some amalgam, he will greatly improve the value of his filling, but will have the discolored tooth nearly or quite as bad as with amalgam.

My experience has led me to mix the zinc and line the cavity well with it, and while plastic press the amalgam into it, clearing the edges so that the zinc will come well to the edge, but so as to be well covered with amalgam. If nicely done, we have no more discoloration than with all zinc. We have the zinc protected from the moisture, and little or no trouble with the edges of our fillings. The zinc becomes a great protection to further decay because of its firmness should moisture pass by the amalgam.—*Items of Interest.*

---

SIMPLE TEST FOR GOOD PLASTER.—The quality of plaster may be tested by simply squeezing it with the hand. If it cohere slightly and keeps in position after the hand has been gently opened, it is good; but if it falls to pieces immediately, it has been injured.—"British Jour. Den. Sci."

VARNISHING CAVITIES.—Do we not get a more lasting filling of amalgam in a tooth, by first varnishing the cavity with a thin solution of rosin in ether?

My method is to,

1st—Isolate tooth by rubber dam;

2d—Remove all decay.

3d—Sterilize cavity by the drug best indicated for case at hand,

4th—Dry cavity thoroughly with alcohol and hot air;

5th—Varnish cavity with thin solution of rosin in ether;

6th—Carefully remove all of varnish from periphery of cavity.

7th—Pack in amalgam in the usual way.

It seems to me in this way we get a more thoroughly incorporated filling.

It is pretty generally allowed by scientists that ether has great penetrating powers; especially is this true in the canaliculi of the tooth structure. By using the rosin and ether, the canalculi become sealed. The amalgam is now pressed in, and a very intimate relation is established between the tooth and filling which would not take place if the varnish were not used.

I have hesitated to fill teeth in this way, because of having had no advice on the subject. I do not claim this as being an original idea.

Perhaps this method is in general use, but if so, I have never heard of it.

I would be pleased to hear the objections to the method, as I am not prejudiced and only want the best method.—GEORGE W. SOULE, in *Items of Intereet.*

---

SUCCESSFUL AMPUTATION OF PULP.—Miss B. presented last month a lower first molar for attention. Upon examination, there was a large mesio-occlusal cavity containing the remains of a cement filling. A gold filling was desired.

During the preparation of the tooth, and in ascertaining the condition of the pulp, I removed the cement and found the tooth did not respond to thermal changes, nor did it show a sensitiveness to excavation or other mechanical influences, and, according to patient's statement, the tooth had not given trouble since it was filled, which was about ten years previous, although at, that time it had caused considerable annoyance.

I drilled into pulp chamber without pain and found no odor. Next, I inserted a broach a third of the way into a canal through what appeared to be the pulp. On reaching to this distance. the patient felt a slight pain and I withdrew the broach.

In a few minutes the tooth was filled with blood. I took the pliers and lifted contents from the pulp chamber, which proved to be a pledget of cotton.

The portion of the pulp contained within the chamber had evidently been extirpated, and the portion in the roots preserved alive.

The operation has been performed at an age when it is reasonable to suppose the roots were not fully developed, and when the foramina had not assumed their normal size; but when I removed the balance of the pulp, I found the roots and foramina perfectly normal, and the tooth in a condition to admit of a successful operation.—H. N. LANCASTER, D. D. S., in *Items of Interest.*

---

CEMENT.—Cement is used a great deal now as a lining under amalgam, when it sometimes serves a triple purpose, protecting the pulp, bracing the walls of the tooth and partial retention. I use it in this way with very great satisfaction. Having given this method some three years' trial, I feel convinced that it is good practice. The qualities necessary for this purpose are the same as for in-lay work. Quickness of setting is especially to be desired. Having coated the cavity walls with the cement, and burnished a layer of amalgam thereon, the cement should set at once to facilitate the removal of the excess from the margin of the cavity, and to prevent oozing around the edges when additional amalgam is added, which is a great annoyance.

In crown and bridge-work the time required for setting is quite a consideration. If a case can be quickly handled, the quicker setting takes place the better, but in some cases it takes a little time to properly adjust a piece of work. Then, of cours, a slower setting material should be used, especially if the weather is warm. For quick work good results may be obtained from Ash's cement, but where much time is required it is better to use something which does no set quickly.—J. W. Bryan, in Western Dental Journal.

---

"WATERPROOF" CEMENTS.—Inasmuch as the durability of a cement filling depends chiefly upon its power to resist attrition and chemical action, it naturally follows that impermeability to moisture is of the utmost importance. A filling which absorbs saliva becomes softer and will certainly wear away faster than one which does not. I proved this to be a fact by the following test: I used two blocks of cement from the same mixture, one of which after thorough setting was subjected to a water bath for one week, the other block being kept dry meanwhile. Each was then subjected to the abrading action of a rather smooth corundum wheel, against which they were held mechanically with a definite pressure, the wheel running of course, at the same rate of speed in each case. This test was tried with several of the non waterproof cements, and in every case a marked difference was shown in the time required for grinding away under the two different conditions. This variation ranged from 35 to 75 per cent.—J. W. Bryan, in Western Dental Journal.

---

AN ALKALIN WASH —The acid exhumation from sore gums is a prolific cause of decay in teeth. Such patients should be impressed with the necessity of the constant use of a mouth-wash. An old-time domestic wash is strong sage tea with borax, sweetened with honey.—"Dental Hints."

STEEL WIRE IN THE MOUTH.—One point I would object to particularly in the paper is the use of a retaining appliance, as suggested by the doctor. He mentioned placing a piece of Stubb's steel wire in the open tubes that are on the adjacent teeth to the one moved. I do not think Stubb's steel wire should ever be placed in the mouth, because its susceptibility to oxidation makes it so irritating that the tissues coming in contact with it are always constantly inflamed. Of course, we can overcome that by plating it, but it is difficult to plate steel, because it has first to be subjected to copper plating, then gold plating, and it is not an easy matter to do it. So I take the stand never to place steel in the mouth, because I do not believe there is any indication for it.—H. J. Goslee, in Review.

---

TO DAM A MOLAR.—To rubber-dam a molar having a grinding surface cavity, and when the dam cannot be forced between the next teeth, affix to the molar a strong clamp and stretch over it and the molar, strong, thick rubber. In most cases the saliva will be dammed out.—Dental Hints.

---

BROKEN BROACH.—Apply a fifty per cent. solution of sulphuric acid to a broken broach or drill in a root canal. Seal in for three or four day, then inject in the root three per cent. pyrozone, washing out the oxid of the metal. With a fine broach the impacted steel is the usually easily removed.—Dental Hints.

